# Pediatric Diabetes Technology Management: An Italian Exploratory Study on Its Representations by Psychologists and Diabetologists

**DOI:** 10.3390/ejihpe13050070

**Published:** 2023-05-21

**Authors:** Annamaria Tupputi, Lucia Giardinieri, Silvia Monaco, Michela Di Trani

**Affiliations:** Department of Dynamic and Clinical Psychology, and Health Studies, University of Sapienza, 00185 Rome, Italy; lucia.giardinieri@uniroma1.it (L.G.); silvia.monaco@uniroma1.it (S.M.); michela.ditrani@uniroma1.it (M.D.T.)

**Keywords:** diabetes, paediatric psychology, health and well-being, health care service, psychology of health, health professionals

## Abstract

The incidence of type 1 diabetes (T1D) has increased by about 3% per year over the last two decades. Continuous Insulin Subcutaneous Therapy (CSII) is widely used in the pediatric population with diabetes; however, it requires more preparation by the treating team and a careful selection of its potential users. Prescriptive provisions vary from region to region, and the perspective of health workers in this regard remains an unexplored area. The aim of this research project is to explore the representations of a group of diabetologists and psychologists working in pediatric diabetology throughout the country, regarding their roles, functions, and activities as part of a multidisciplinary team; it also aims to investigate their views on the potential benefits of CSII and the types of individuals who apply for the use of this technology. A socio-anagraphic data sheet was administered, and two homogeneous focus groups were conducted, one for each profession, which were then audio recorded. The transcripts produced were analyzed using the Emotional Text Mining (ETM) methodology. Each of the two corpora generated three clusters and two factors. For diabetologists, a focus on patient care emerged at different levels, involving collaboration with other health professionals and engagement with the community, often incorporating technology in medical interventions. Similarly, psychologists’ representations highlighted interdisciplinary networking with a stronger emphasis on the psychological processes involved in managing the disease, from acceptance to the elaboration and integration of diabetes into the family narrative. Understanding the representations of health professionals working in pediatric diabetes with new technologies can contribute to the consolidation of a network of professionals through targeted work on possible critical issues that may arise.

## 1. Introduction

Type 1 diabetes (T1D) is one of the most common metabolic and endocrine conditions in childhood, with an incidence rate that has increased by 3% per year over the last two decades [1]. According to the data from the National Institute of Health for the five-year period 2005–2010 [2], the national average incidence rate of T1D in children aged between 0 and 4 years is 13.4 per 100,000 per year. The incidence rate is higher in boys (14.1 per 100,000 per year) than in girls (12.7 per 100,000 per year) [3].

Continuous subcutaneous insulin infusion (CSII), especially in the pediatric population with diabetes, is widely used to achieve better dosing accuracy and flexibility in insulin regulation [4]. There has been a consistent increase in the number of patients transitioning from multi-injection therapy to CSII. The International Society for Pediatric and Adolescents recommends CSII as the primary treatment choice for preschool children [5,6]. The publication of national guidelines for the use of CSII [7] and the introduction of increasingly sophisticated insulin pumps in the Italian market may have further contributed to the preference of caregivers and patients for CSII [8]. Despite the scientific evidence supporting its use, implementing CSII requires extensive preparation and education from the treating team, as well as careful selection of suitable candidates for this technology [9].

The optimal organization of CSII therapy management should involve dedicated clinics and staff, a multidisciplinary team with expertise in the field, and the provision of 24-h contact with the patient through a dedicated telephone line [7].

Therapeutic education should be conducted by a properly trained and multidisciplinary team capable of considering the specific needs of children and their families. Children with diabetes differ from adults in several aspects, including their insulin sensitivity during sexual maturation, their ability to practice self-management, the variability of their eating behaviors, and their physical activity. It is also crucial to pay attention to family dynamics, developmental stages, and psychological differences related to sexual maturity [7]. Therefore, the training of the treating team should be carefully planned, continuous, and repeated, focusing on educational strategies and effective communication with both the child and the family [10].

In Italy, there is currently no National Register in place, and only a minority of the surveyed facilities meet all these requirements. A significant number of facilities still lack a dedicated clinic for CSII therapy and do not have a multidisciplinary team that includes a diabetologist, nurse, dietitian, and, whenever possible, a psychologist. These services should aim to be safe, effective, patient-centered, accessible, and well-organized. They are intended to facilitate assistance and reduce healthcare costs associated with disease complications. The planning of work activities, combined with the workplace’s climate, organizational culture, and interpersonal relationships, contribute to a complex emotional context that healthcare workers are exposed to in their professional setting [11]. In a study [12], healthcare professionals were found to perceive limited professional development opportunities and a lack of adequate training, which in turn leads to uncertainty about their work and a decrease in self-esteem. These limitations in work and organizational resources have a direct impact on the quality of care provided to patients. To enhance the effectiveness of healthcare services, there is a need for programmatic and social policies that prioritize stress prevention and promote the empowerment of healthcare professionals [12]. In the literature, predominantly quantitative methodologies have been employed to investigate the attitudes of physicians and nurses towards patient autonomy, the multidisciplinary approach to diabetes care, and the psychosocial impact of the disease [13,14]. However, some researchers [15] have adopted a qualitative approach to understand professionals’ perspectives on the use of CSII and strategies for identifying patients who would benefit most from this technology. This approach highlights the importance of exploring practitioners’ views to uncover attitudes or misconceptions that may influence patient selection when considering CSII. Qualitative methodologies have been used with children [16,17], and in various countries—including Ireland [18], the UK [15,19], and New Zealand [20]—to examine healthcare workers’ attitudes and opinions on this matter.

Qualitative methods can be useful to explore topics on which the literature is limited [21] and/or in community-based research projects when engagement and the involvement of multiple stakeholders are goals [21]. In the field of this article, qualitative methodologies allow the researcher to understand the various factors involved in pediatric primary care by integrating the perceptions of a wide range of stakeholders concerning behavioral and mental health needs and care [22,23]. In this way, it is possible to reflect on what is working, what is not, what is confusing, and what is important [24,25].

Particularly, focus groups enable researchers to quickly identify participants’ perspectives [22,26]. Due to the interactional nature of focus groups, participants can clarify and expand their contributions by comparing with the points of view of the other participants.

However, research related to the involvement of stakeholders in studying mental health in pediatric primary care is still lacking [27,28]. In Italy, no studies of this nature have been conducted, resulting in a significant gap in the literature. Each country has its own distinct culture, which can manifest in variations within micro-groups such as professionals working with pediatric diabetes and CSII. These differences can be attributed to the emotional representations that characterize specific groups of individuals [29]. These representations influence individuals’ attitudes and emotions surrounding the topic, and consequently, their behaviors and actions. Therefore, it is crucial to investigate the emotional representations of professionals dealing with this topic in Italy, in order to uncover the underlying culture that characterizes this field.

The aim of this research project is to explore the representations of Italian diabetologists and psychologists regarding pediatric diabetes, technologies such as CSII, and the potential differences between these two professional groups.

## 2. Materials and Methods

### 2.1. Sampling and Recruitment

To investigate the functions and roles of Italian diabetologists and psychologists working with pediatric diabetes, a total of 53 Italian pediatric diabetes centers were invited via email. Specifically, critical recruitment criteria were applied for psychologists (PSY) and diabetologists (DIAB), which included having a minimum of one year of work experience with patients with T1D and being part of the same team.

Out of the 53 centers, 37 did not respond, 4 did not meet the criteria, and 7 were unavailable. Five centers agreed to participate in the research, resulting in a total of 11 participants (5 DIAB and 6 PSY; as shown in Table 1). The majority of the participants were women in the age group of 46–55 years, and most of them were from northern Italy. The professionals were divided into two homogeneous focus groups based on their profession.

The participants were asked three specific questions:

- What is your role within the multidisciplinary team in the field of pediatric diabetology?
- What emotions do you experience when using technological devices in the therapy of pediatric patients? How do you interact with their families?
- What factors influence your recommendation for or against the use of technological devices such as CSII?

### 2.2. Data Collection

The diabetes centers were contacted between March and April 2022, and the focus groups were conducted in May 2022. Each group session lasted approximately an hour and a half. Prior to participating in the research, every participant signed an informed consent form and completed a sociodemographic questionnaire, providing details such as their gender, age, occupation, and so on. To adhere to the COVID-19 safety measures that were in place, the focus groups were conducted via video call, facilitated by two experienced psychologists. The conversations were audio recorded, transcribed, and completely anonymized. The same topics were discussed in both focus groups.

### 2.3. Data Analysis

The focus groups were recorded and transcribed in their entirety. The transcriptions were organized into two small corpora: one for DIAB (with a token count of 5292) and one for PSY (with a token count of 8391). To explore the emotional representation of these professional groups in the management of pediatric diabetes technology, we utilized the Emotional Text Mining (ETM) methodology [29,30]. ETM is a non-supervised text mining procedure that draws on a psychodynamic model and a socio-constructivist approach [31]. It enables the identification of both the semantic and semiotic aspects conveyed through communication. The semantic level focuses on the content of the communication, while the semiotic level pertains to the emotional symbolization embedded within the communication. Individuals emotionally symbolize events or objects and socially share these symbolizations, which can influence behaviors, expectations, and interactions through unconscious mental processes [29,30,32]. ETM utilizes a statistical procedure that simulates the thinking process, starting from the semantic level (conscious) and progressing to the semiotic level (unconscious), contrary to the human thinking process [33]. To achieve this, ETM employs a sequence of synthesis procedures that involve stem type reduction, keyword selection, and extension into cluster and factor analysis. These steps aim to identify the semiotic-symbolic level based on the semantic matrix [30].

To assess the feasibility of applying ETM to the data, two lexical indicators were calculated for each corpus: the type-token ratio and the percentage of hapax. The results were as follows: DIAB: TTR = 0.234 and Hapax = 60.4%; PSY: TTR = 0.225 and Hapax = 57.0%. These findings align with the small size of the corpora [34]. The corpora of the two transcriptions were initially cleaned, preprocessed, and subjected to keyword selection to detect associative links between words, infer the symbolic matrix, and determine their coexistence within the texts. A cluster analysis was conducted using a bisecting k-means algorithm based on cosine similarity on the text-keyword matrix, with a maximum of ten partitions. Texts that did not include the co-occurrence of at least two keywords were excluded. To identify the optimal clustering validation measure, we considered the Calinski-Harabasz, Davies-Bouldin, and intraclass correlation coefficient indices, which are commonly used in text mining procedures [35]. Subsequently, a correspondence analysis [36] was performed on the cluster per keyword matrix to identify the symbolic matrix. The four investigators independently interpreted the factorial space based on word polarization [29], assigning labels to each factor and polarity. The clusters were then interpreted based on their location in the factorial space, the words characterizing the context units classified within the clusters, and the identified representation. The labels were analyzed and discussed to define the final interpretation and labeling.

## 3. Results

The results of the analysis showed that the keywords selected allowed the classification of 99.05% (DIAB) and 97.02% (PSI) of the content units. The clustering validation measures showed that the optimal solution was three clusters and two factors for both groups of professionals.

### 3.1. Factors

As shown in Table 2, DIAB participants represented professionals working in multidisciplinary teams of Italian pediatric hospitals through two main symbolic categories, “Taking Charge” and “Healthcare”. Each factor is composed of two polarities, which contain a series of representative words.

The first factor highlights the diabetologist’s “Taking Charge”. This emotional dimension is characterized by the Team (negative polarity), in which diabetologists are part of an healthcare facility from a teamwork perspective, and by Relationship (positive polarity), in which the therapeutic relationship is expressed through an operating mode. In the Team, the relationship with the patient is not mentioned, but emphasis is given to the team network, while in Relationship, the relationship is played by the lonely diabetologist who face the family and the decision to use new technologies.

The second factor focused on “Healthcare”, highlighting two poles, Management and Care. On the one hand (Management) there is a dimension of coordination, a team that manages the diagnostic and therapeutic process, from which, however, some figures are excluded, those who have direct contact with the patient, or those who deal with Care.

With regards to psychologists’ results (Table 3), the PSY participants represented their working in the same context through the two other main symbolic categories: “Psychologist’s Work” and “Levels of intervention”.

The first factor of PSY highlights the Psychologist’s Work, which is characterized by the process (negative polarity) and its steps (from the diagnosis to the acceptance of diversity, impotence, and the treatment), and by the network (positive polarity) that includes all the actors and contexts that relate daily to the pathological reality of the patient.

The second factor focused on Levels of intervention, with the negative pole, Processing the pathology, and the positive pole, Acceptance of the pathology. In the first one, the emotional component linked to the onset of the pathology emerges, and so do the changes that it involves in the patient’s life; the second one highlights the experiences linked to the path of care and the acceptance of the technology that are in the relationship between the psychologist and patient.

### 3.2. Cluster

#### 3.2.1. Diabetologists

Based on the results of the DIAB group (Table 4), the first cluster (Table 5), named Response to the need (38% of Unit Contexts), reflects the support and assistance that the different actors of care—from the health professionals to the territorial context—offer to the patient.

To support the cluster analysis, we decided to insert quotes following the TF-IDF score of Salton [37], a measure useful in estimating the importance of a lexical unit in a document:

Score (51.828): “In my opinion, having a dietitian present in the clinic, as my colleagues described in their experience, is crucial. While calling a dietitian during a visit, which you cannot dedicate more than 20 min to, you have to identify the problem and call the dietitian, who may be somewhere else dealing with metabolic diseases”.

Original quote in Italian: “Secondo me la presenza in ambulatorio, come raccontavano le colleghe prima della loro realtà, di avere una dietista sempre presente è fondamentale. Mentre a chiamata, nel contesto della visita, alla quale poi non riesci a dedicare più di 20 minuti, devi cogliere il problema, chiamare la dietista, la dietista magari è da un’altra parte che fa le malattie metaboliche”.

The second cluster, named Pathological reality (24% of U.C.), contains everything concerning the management of the disease by a predominantly medical team (only the name of the pediatrician appears, while the other professional figures, such as the dietician and the psychologist, appear in the first cluster). The cluster makes us reflect globally on the degree of real or imaginary conjunction existing between the different professionals within an interdisciplinary work. In addition, a key figure, the patient, is not mentioned.

Score (35.823): “I connect with what my colleague said: it is easy to work with those who are good, with those who accept technology, with good families—very easy to work with those. Once a colleague from xxx said, ‘save one of those that goes wrong’… That is your job, your satisfaction”.

Original quote in Italian: “Mi riallaccio a quello che diceva il collega, è facile lavorare con quelli che vanno bene, con quelli che accettano la tecnologia, con le famiglie brave, facilissimo lavorare con quelli, una volta un collega di Modena disse: salvare uno di quelli che va male… quello è il tuo lavoro, la tua soddisfazione”.

The third cluster, Family meets Technology, (38% of U.C.) underlines the meeting between the nucleus of the family and the technology (the pump), where the diabetologist is a spectator. There seems to be a request for help, as the professional seems to be frozen and passive in this scenario.

Score (35.257): “So obviously the patient’s choice is essential, but there is a variety of attitudes from a technological perspective, like whether to use an insulin pump or not, with different preferences for the person, the child, and the family… Sometimes the family wants it but the child doesn’t, and vice versa”.

Original quote in Italian: “Quindi ovviamente la scelta del paziente è essenziale però c’è una varietà di atteggiamento dal punto di vista della tecnologia, microinfusore si, microinfusore no, con tempi diversi della persona, del bambino, della famiglia… a volte c’è una famiglia che lo vuole e il bambino no e viceversa”.

#### 3.2.2. Psychologists

With regards to the three clusters of the PSY group (Table 6), the first one (Table 7), named Networking (45% U.C.) includes the spirit of fellowship, a team-work that monitors the different levels of work with the patient. The aim of this work is the acceptance of the pathology and of the treatment.

Score (34.554): “As my colleague from XXX said, the interface and balance between hospital and community care is always very delicate in a chronic condition that only comes to the hospital for a few days of admission and is mainly managed in the community. However, where we are, community intervention is mainly related to school-related activities”.

Original quote in Italian: “Come diceva la collega di XXX è sempre molto delicata l’interfaccia, l’equilibrio ospedale/territorio in una patologia cronica che di fatto arriva in ospedale soltanto per i pochi giorni del ricovero e che si esplica, si vive tutta sul territorio, ma laddove qui da noi l’intervento del territorio è prevalentemente legato agli interventi di tipo scolastico”.

The second cluster, Working with the patient (17% U.C.), refers to a more mature process of pathology management, including the acceptance of the disease and the feeling of impotence and the elaboration of the cure process.

Score (53.277): “Then this episode, gestational diabetes regresses, puts at risk type 2 diabetes in the future; however, the sense of diversity, when they told me about this diversity and then the sense of impotence, I am trying to help the people who are fighting, because it is not a war—it cannot be a war”.

Original quote in Italian: “Allora questo episodio, il diabete gestazionale regredisce, pone a rischio di diabete tipo 2 nel futuro però, il senso di diversità, quando mi parlavano di questa diversità e poi il senso di impotenza, di una lotta, tanto che adesso anche negli interventi cerco di aiutare le persone che lottano contro, perché non è una guerra, non può essere una guerra”.

The third Psychologists cluster, Working with the family system (38% U.C.), alludes to the attention to the patient and his family: the work implies the attempt to integrate the event of diabetes within the family narrative.

Score (32.719): “It’s as if a child is born, as if there is a new one, someone tells us about before and after the onset, as if before the onset was the year 0. The onset and from there we start again, there is a new life, everything has changed and time, their life stands out precisely on a before and after. This because it marks, is a moment that marks particularly.”

Original quote in Italian: “È come se nascesse un bambino, come se ci fosse una nuova, qualcuno ci parla di prima e dopo dell’esordio, come se prima dell’esordio fosse l’anno 0. L’esordio e da lì si riparte, c’è una nuova vita, tutto è cambiato e il tempo, la loro vita si distingue proprio su un prima e il dopo. Questo perché segna, è un momento che segna particolarmente”.

## 4. Discussion

The aim of the present study was to identify the emotional representations surrounding the topic of pediatric diabetes and the use of related technologies in a specific group of professionals, namely diabetologists and psychologists. To achieve this goal, two profession-specific focus groups were conducted, and the transcripts were analyzed using the ETM methodology. Each group yielded three clusters and two factors that described the emotional dimensions and representations of each profession regarding the investigated topic. Diabetologists exhibited an emotional space characterized by two dimensions: Taking Charge (between the Team and Relationship) and Healthcare (between Management and Care). Psychologists, on the other hand, demonstrated dimensions of Psychologist’s Work (between the Process and the Network) and Levels of Interventions.

It is interesting to note that the first factor in both groups focuses on the type of work performed for the patient. Psychologists describe a dimension of network and territory work, while diabetologists emphasize teamwork. Although the psychologist is present in the team represented by the diabetologist, this dimension does not emerge in the psychologists’ results themselves. These findings likely indicate a difficulty for psychological professionals to effectively integrate into work teams within this particular context. However, the presence of this representation within the diabetologists’ factors suggests a starting point for building a team where the competence and psychological expertise are effectively present and actively contribute to teamwork, rather than solely focusing on territorial aspects. Additionally, within the first factor of diabetologists, there is a relational dimension where the diabetologist is somewhat “excluded,” and the family’s decisions regarding new technologies appear as a challenging and impenetrable context for the diabetologist. This aspect could serve as a meeting point within the team where the psychologist can not only address the “need” but also emotionally support the diabetologist in understanding the patient and their family’s experiences.

Furthermore, the second factor related to the Care component of the diabetologists is limited, suggesting a difficulty in adopting this dimensional perspective. A psychological component could support a vision of care that encompasses not only management but also actual care.

Another significant finding is the importance, for both professionals, of working with the family rather than solely focusing on the individual. This applies to both accepting and managing the illness and incorporating new technologies. To ensure the success of a treatment involving the use of technologies, it is essential to consider the family system and provide support from preparation to ongoing assistance throughout the process. Teamwork and networking also play a crucial role in this context, indicating that the issue of pediatric diabetes and the use of related technologies should be approached from a network perspective involving not only individual professionals but also groups of experts or individuals who can impact the final outcomes. Meeting the needs, therefore, requires a team-based approach with a focus on comprehensive care and particular attention to the territorial network.

To this end, establishing a network of professionals at the local level and providing specific training to practitioners who can adopt a family-oriented approach and collaborate with other professional figures appears crucial.

The literature has demonstrated that focus groups serve as actual psychological interventions and have an impact through the re-elaboration of concepts and the exchange of different perspectives among participants [38,39]. Therefore, we consider this experience as a genuine intervention for the participants, and we hope that it has helped the professionals involved to develop a broader perspective that recognizes the importance of working within a network. The medical team plays a significant role in the complex comprehensive care system for pediatric patients [40].

## 5. Limitations

This research has several limitations. Firstly, there was a low response rate from the Italian Pediatric Diabetes Centers that were contacted, resulting in a small sample of participants. This is a common challenge in qualitative research, where participants are invited to play an active role in focus groups. For future studies, it is important to establish a strong network for participant recruitment and physically present the research in the centers to enhance engagement levels.

Furthermore, it would be beneficial to further investigate the use of the same methodology (ETM) in exploring the emotional representations of patients and their families regarding pediatric diabetes and its technology. For instance, ETM could be valuable in exploring the various possible dimensions of diabetes in pediatric patients, allowing for focused interventions that consider the representations and attitudes of young patients towards diabetes management [41,42]. Additionally, it would be highly interesting to apply the same investigative methodology to another crucial sample in the context of pediatric diabetes and its management: nurses.

At last, this study aims to promote the future construction of intervention strategies involving the various community agencies of the pediatric diabetes patient management. Multi-sector partners, such as Academic, Public Health, Private Foundations and Associations could be set up to build shared national guidelines, reducing disparities in health service provision [42].

## Figures and Tables

**Table 1:** Demographics variables of participants.

	*n (%)*
Gender	
Male	1 (9.1%)
Female	10 (90.9%)
Age Group	
35–45	3 (27.3%)
46–55	7 (63.6%)
56–65	1 (9.1%)
Education Level	
Specialization	9 (81.8%)
PhD/Master	2 (18,2%)
Type of Profession	
Diabetologists	5 (45.4%)
Psychologists	6 (54.6%)
Area of Origin	
North of Italy	9 (81.8%)
Centre of Italy	2 (12.2%)
South of Italy	0 (0.0)
Years of service	
0–10	4 (36.4%)
11–20	5 (45.4%)
21–30	1 (9.1%)
31–40	1 (9.1%)

**Table 2:** Diabetologists’ factors 1 e 2 emerged from the analysis. Keywords are presented by order of percentage of absolute contribution (CA%).

Factor 1 Taking Charge				Factor 2 Healthcare			
Team	CA%	Relationship	CA%	Management	CA%	Care	CA%
Nurse Dietitian Surgery Psychologist Department Activity Association	−6.45 −5.61 −5.05 −3.33 −2.8 −1.68 −1.4	Technology Adolescents I think Choosing Put Pump Parent	9.37 6.55 4.54 4.54 4.03 4.03 2.92	Work Occupying Pediatrician Maintain Team Relation diabetes	−12.69 −6.82 −6.82 −5.45 −4.87 −3.65 −3.53	Nurse Dietitian Surgery Patients Ward	3.63 3.15 2.84 1.82 1.58

**Table 3:** Psychologist’s factors 1 e 2 emerged from the analysis. Keywords are presented by order of percentage of absolute contribution (CA%).

Factor 1 Psychologist’s Work				Factor 2 Levels of Intervention			
The Process	CA%	The Network	CA%	Processing the Pathology	CA%	Acceptance	CA%
Accept Put Diversity Present Happen Sense Impotence Immediately	−5.89 −5.58 −4.13 −2.95 −2.75 −2.62 −2.36 −1.77	School Colleague Glycemia Mum Team Hospital Work Territory	4.34 4.34 2.31 1.96 1.72 1.7 1.67 1.58	Onset Lived Augment Person Anger Think Patient New	−3.13 −2.34 −2.19 −2.19 −2.19 −2.06 −1.91 −1.87	Put Accept Diversity Small Present Pump Impotence Creed	7.26 5.95 4.16 2.97 2.97 2.46 2.38 1.91

**Table 4:** Diabetologists cluster location in the symbolic space (under the factor polarity interpretation, the cluster coordinate of each factor is reported).

Cl	%UC	Factor 1 Taking Charge	Factor 2 Healthcare
1 Response to the need	38%	Team (−0.7)	Care (0.4)
2 Pathological reality	24%	Team (−0.1)	Management (−1.1)
3 Family meets Tecnhology	38%	Relationship (0.8)	Care (0.2)

**Table 5:** The three clusters of Diabetologists.

Cluster	Label	Keyword
1	Response to the need	Nurse Dietitian Surgery Psychologist Department Activity Need Association
2	Pathological reality	Work Diabetes Reality Pediatrics Team Occupying Pediatrician
3	Family meets Technology	Technology Adolescents Family Parent I think Choosing Onset Pump

**Table 6:** Psychologists cluster location in the symbolic space (under the factor polarity interpretation, the cluster coordinate of each factor is reported).

Cl	%UC	Factor 1 Psychologist’s Work	Factor 2 Levels of Intervention
1 Networking	44.79	The network (0.6)	Acceptance (0.2)
2 Working with the patient	17.18	The process (−1.1)	Acceptance (0.9)
3 Working with the family system	38.04	The process (−0.3)	Psychological processing of the pathology (−0.6)

**Table 7:** The three clusters of Psychologists.

Cluster	Label	Keyword
1	Networking	Colleague School Territory Blood sugar Hospital Mom Team
2	Working with the patient	Put Accept Pump Diversity See Small Present
3	Working with the family system	Debut Think about Parent Household Patient Lived Life

## Data Availability

Data available by the authors without undue reservation.
